# Oversulfated Chondroitin Sulfate Binds to Chemokines and Inhibits Stromal Cell-Derived Factor-1 Mediated Signaling in Activated T Cells

**DOI:** 10.1371/journal.pone.0094402

**Published:** 2014-04-09

**Authors:** Zhao-Hua Zhou, Elena Karnaukhova, Mohsen Rajabi, Kelly Reeder, Trina Chen, Subhash Dhawan, Steven Kozlowski

**Affiliations:** 1 Division of Monoclonal Antibodies, Center for Drug Evaluation and Research, Food and Drug Administration, Bethesda, Maryland, United States of America; 2 Division of Hematology, Center for Biologics Evaluation and Research, Food and Drug Administration, Bethesda, Maryland, United States of America; 3 Division of Therapeutic Proteins, Office of Biotechnology Products, Office of Pharmaceutical Science, Center for Drug Evaluation and Research, Food and Drug Administration, Bethesda, Maryland, United States of America; 4 Division of Emerging and Transfusion Transmitted Diseases, Center for Biologics Evaluation and Research, Food and Drug Administration, Bethesda, Maryland, United States of America; University of Leicester, United Kingdom

## Abstract

Oversulfated chondroitin sulfate (OSCS), a member of the glycosaminoglycan (GAG) family, was a contaminant in heparin that was linked to the 2008 heparin adverse events in the US. Because of its highly negative charge, OSCS can interact with many components of the contact and immune systems. We have previously demonstrated that OSCS inhibited the complement classical pathway by binding C1 inhibitor and potentiating its interaction with C1s. In the present study, by using surface plasmon resonance, we found OSCS interacts with T cell chemokines that can impact adaptive immunity. The binding of OSCS to stromal cell-derived factor-1 (SDF-1) chemokines, SDF-1α and SDF-1β, caused a significant change in the secondary structures of these chemokines as detected by far-ultraviolet circular dichroism spectra analysis. Functionally, OSCS binding profoundly inhibited SDF-1-induced calcium mobilization and T cell chemotaxis. Imaging flow cytometry revealed T cell morphological changes mediated by SDF-1α were completely blocked by OSCS. We conclude that the OSCS, a past contaminant in heparin, has broad interactions with the components of the human immune system beyond the contact and complement systems, and that may explain, in part, prior OSCS-related adverse events, while suggesting potentially useful therapeutic applications for related GAGs in the control of inflammation.

## Introduction

Oversulfated chondroitin sulfate (OSCS), a contaminant in heparin linked to the 2008 heparin adverse events in the United States, has become the subject of multidisciplinary investigations [1], [2]. Initially, OSCS was found to cause the activation of contact system (also known as the intrinsic pathway of coagulation or kallikrein/kinin system [3]) through binding with factor XII and generation of plasma kallikrein and bradykinin, as well as the anaphylatoxins C3a and C5a [4], [5]. OSCS also interacts with other parts of the innate immune system including most of the elements in the classical complement pathway [6]. We have previously reported that OSCS inhibited complement fixation on bacteria and complement-mediated bacterial lysis by potentiating C1 inhibitor activity [1]. Surface plasmon resonance assays showed OSCS has stronger binding than chondroitin sulfate A (CSA), its less sulfated progenitor, and heparin to a variety of plasma proteins including FXII, complement components C1 to C9, and C1inh [1], [6]. Since heparin can bind a variety of chemokines [7], important components of the immune system, it is of interest to evaluate if OSCS also interacts with chemokines. Assessing the nature of such interactions can enhance our understanding of the physiological and pathological roles of GAGs in the regulation of innate and adaptive immunity.

Chemokines are a family of small cytokines between 8–10 KD secreted by a variety of cell types [8]. Chemokines bind to cell-surface G protein-coupled receptors and transmit signals that are critically involved in many biological processes [9], [10]. One of the most important and studied chemokines is SDF-1, also named CXCL12. SDF-1 belongs to the CXC subfamily of chemokines characterized by the presence of four conserved cysteines, which form two disulfide bonds. SDF-1 had been reported to be produced in two forms, SDF-1α/CXCL12a and SDF-1β/CXCL12b, by alternate splicing of the same gene [11] and more recently, six isoforms of SDF-1 were identified in humans [12], three (α, β, γ) of which have been investigated for the binding to heparan sulfate (HS) [13].

The binding of SDF-1 with CXCR4, its primary receptor, plays important roles in lymphocyte trafficking, cancer metastasis, bone formation, embryonic development and pathogenesis of HIV/AIDs [14]–[21]. To function properly, chemokines require interaction not only with G protein–coupled receptors (GPCRs) but also with the glycosaminoglycan (GAG) carbohydrate moieties (e.g., heparan sulfate) of proteoglycans on endothelial cells and the extracellular matrix [7]. This low avidity non-covenant binding maintains the chemokine gradients that are necessary for cell trafficking. The chemokine gradient can be interrupted by higher affinity binding GAGs [22], such as heparin. Heparin can disrupt the SDF-1/CXCR4 axis and impair the functional capacity of bone marrow-derived mononuclear cells involved in cardiovascular repair [23].

Structurally, OSCS belongs to the family of GAGs, which includes heparin, heparan sulfate, dextran sulfate, chondroitin sulfate A (CSA), chondroitin sulfate -B, C, E and their oversulfated forms, characterized by large linear polysaccharides constructed of repeating but heterogeneous disaccharide units: a combination of an uronic (glucuronic or iduronic) acid and an amino sugar (*N-acetyl*-D-glucosamine or *N-acetyl*-D-galactosamine) [24]. OSCS is semi-synthesized from CSA and has a distinct structure with additional sulfonated groups as compared to heparin or other GAGs [2]. Oversulfated chondroitin/dermatan chains have been demonstrated to inhibit chemokine activity in vitro [25]. Therefore, the interaction of the highly sulfated OSCS with chemokines is important to study. This investigation may not only reveal potential mechanisms of OSCS-induced heparin-associated clinical AEs, but may also provide new strategies for altering chemokine function with GAGs in the treatment of chemokine-involved diseases.

## Materials and Methods

### Ethics Statement

Human peripheral blood mononuclear cells were obtained by leukapheresis of normal volunteers from the Department of Transfusion Medicine, NIH with informed consent under NIH Institutional Review Board approval. The use of these human cells without identifiers was approved as an exemption that covers this study by the Institutional Review Board of the Food and Drug administration, Silver Spring, MD.

### Materials

OSCS-contaminated and un-contaminated heparin lots were obtained by the FDA from Baxter Healthcare (1000 U/ml or 5000 U/ml in 10 ml and 30 ml vials) during the 2008 heparin crisis. The contaminated heparin contains around 20% OSCS and the un-contaminated lot is OSCS-free [26] [27]. Synthetic OSCS was obtained from the Division of Pharmaceutical Analysis, FDA at St. Louis. Chondroitin sulfate A (CSA) was purchased from Sigma (St. Louis, MO). Recombinant human chemokines were purchased from Cell Sciences (Canton, MA). FITC anti-human CD43 monoclonal antibody and 7AAD cell viability solution were purchased from BD Biosciences (San Jose, CA). Draq5 was purchased from AXXORA, LLC (San Diego, CA). Aamine-PEO3-biotin was obtained from Pierce (Rockford, IL); Biotinylated GAGs were prepared as described by Li et al [28]. Fluo-4, Fura red dye, and F127 were purchased from Molecular Probes, Inc. (Eugene, OR).

### SPR Measurements of Chemokines Binding to Immobilized CSA, Heparin and OSCS

The streptavidin sensor chips (GE Healthcare, Uppsala, Sweden) were pretreated with three 5 μL injections of 50 mM NaOH in 1 M NaCl, to remove any nonspecifically bound contaminants. 20-μL of biotinylated CSA, heparin or OSCS (500 μg/mL) in HBS-PE+ running buffer (flow rate, 10 μL/min) were injected in flow cells 2, 3 and 4, respectively until the targeted RU (200 to 800) was reached, followed by a 10-μL injection of 50% isopropanol/50 mM NaOH/1 M NaCl for 30 s. Flow cell 1 was similarly treated with buffer in the absence of biotinylated GAGs (control).

SPR was performed on a BIAcore T200 (GE Healthcare). Chemokines were diluted in HBS-EP+ buffer (GE Healthcare). Chemokine dilutions were injected at a flow rate of 20 μL/min. After sample injection (120 s), dissociation was evaluated for 180 s. The sensor surface was regenerated by injecting 50 mM NaOH in 1 M NaCl for 30 s. The response was monitored as a function of time (sensorgram) and analyzed by Biacore T200 Evaluation software.

### Circular Dichroism

CD measurements were performed on a Jasco J-815 Spectropolarimeter (JASCO Co., Japan) at 25±0.2°C. Each far-UV CD spectrum was averaged from five consecutive scans over the range of 180–260 nm in a quartz cuvette with 1 mm pathlength using the same measurement parameters: a scan speed of 50 nm/min, bandwidth of 1.0 nm, and resolution of 0.2 nm. The protein concentration was 12.5 μM in PBS, pH 7.4. The baseline was subtracted by running PBS as a blank. The ellipticity of CD spectra was expressed in millidegrees (mdeg). For evaluation of the secondary structure elements, the initial CD spectra of SDF-1α and SDF-1β samples (in mdeg) were converted into Δε per amino acid residue and analyzed using CDPro/CONTIN. Protein samples were titrated with heparin, CSA or OSCS by adding small increments (0.5–1 μL) of a 1 mM stock solution of each GAG in double distilled water.

### Human Peripheral Blood T cells and T cell Blasts

Freshly isolated human peripheral blood T cells were cultured at 1×10^6^/ml in DMEM containing 10% FCS, 10 units/ml rIL-2 and 1% PHA (Invitrogen, Grand Island, NY). The culture medium was changed every other day. On day 3, the T cell blasts were washed with serum free medium twice and re-suspended in serum-free medium for further use.

### Chemotaxis

T cell blasts were loaded with Draq5 at 0.5 μl/10^6^ cells for 20 mins. 10^5^ cells in 100 μl of serum-free medium were loaded in triplicate into the upper chambers of 24-well transwell plates (Corning Inc., Corning, NY). The lower chambers were loaded with serum-free medium (Invitrogen), and chemokines with or without GAGs. The chemokine concentration for maximum migration was determined (100 ng/mL for SDF-1). After incubation for 2 h at 37°C, cells from the lower chambers were vigorously re-suspended and counted by flow cytometry using BD Trucount tubes (BD Biosciences). Draq5 positive cells were counted to exclude cell debris.

### Calcium Influx Assay by Flow Cytometry

1×10^7^ T cell blasts were re-suspended in 1.5 mL Buffer 1 (HBSS containing 0.5 mM MgCl_2_, 1 mM CaCl_2_, and 0.1% BSA). 10 μg Fluo-4 and Fura red dye were added together with F127 (mixed and dissolved in 10 μl DMSO), and the cells were incubated at 37°C for 30 mins. The cells were then washed twice with Buffer 1 and re-suspended with 3 mL of Buffer 1 containing 60 μl of 7AAD, aliquoted and kept on ice. An LSRII flowcytometer (BD Biosciences) was set with the following parameters for data collection: FSC, SSC, Time, 530/30 (Fluo-4, blue laser), 695/40 (Furo red, blue laser) and 660/20 (7AAD, green laser). Aliquots of T cells were warmed at 37°C for 5 mins before loading into the flow cytometer. Cells were collected (1000–2000 cells/s) for 30 s as a baseline, at which time 50 μl of control (HBSS), SDF-1 or SDF-1 in the presence of GAGs were injected. The final concentration of SDF-1 was 100 nM (∼800 ng/mL), and the final concentration of the GAG was 10 μg/ml. The calcium flux [29] was analyzed using FlowJo software (TreeStar Inc., Ashland, OR). 7AAD was used to exclude dead cells and the kinetics of Fluo-4/Fura red ratio is shown.

### Imaging Flow Cytometry Analysis for Cell Morphological Changes

Cell morphological changes were determined using the ImageStream system (Amnis Corporation, Seattle, WA). 1×10^7^ T cell blasts were stained with anti-CD43-FITC, Draq5 (10 μl) and 7AAD for 30 mins at 4°C. The cells were then washed twice and re-suspended in serum-free medium. An aliquot (1×10^6^) of cells in 50 μl serum-free medium were brought to 37°C, injected with 50 ng/10 μl of SDF-1 (with or without 1 μg GAGs), and then the cells were set at 37°C for 10 mins before loading onto the ImageStream. Cell images (>10,000), were collected at ∼50 cells/s with the IS100 ImageStream system and analyzed using ImageStream Data Analysis and Exploration Software (IDEAS, Amnis Corporation). Single fluorochrome-binding cells were collected and used for fluorescence compensation. The circularity feature with membrane CD43 staining was used to analyze the morphological changes of T cell blasts after chemokine stimulation. A single cell (based upon Draq5 staining), live (7AAD negative), focused, and CD43 positive population was gated. Circularity scores for the gated cells in each sample were plotted in a histogram and a low circularity population was identified.

### Statistical Analysis

Duplicate or triplicate samples were evaluated and the experiments were repeated at least three times. The data are presented as mean or mean ± standard error of the mean. ANOVA tests were run for multiple groups in the SDF-1 mediated chemotaxis study and significance was set at a 95% confidence interval. All the paired comparisons were subjected to two-tailed Student’s t tests. Significance was set at a p value of less than 0.05. All differences noted in experiments with multiple paired comparisons were significant at a p value of <0.01. For the ImageStream circularity analysis, paired comparisons were significant with Student T critical values that correspond to p values between 0.01 and 0.02.

## Results

### OSCS Binds to Chemokines

**Figure 1:**
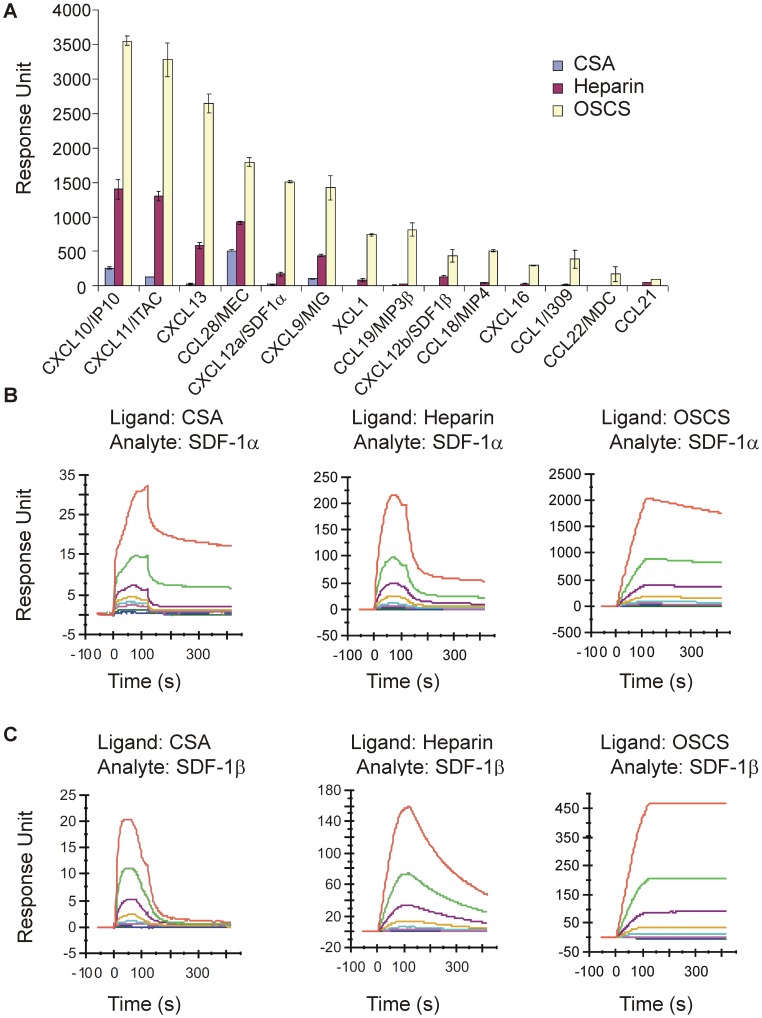
Comparison of GAG binding with chemokines. The biotin-labeled GAGs, CSA, heparin (OSCS-free) and OSCS, were immobilized on a streptavidin sensor chip as described in Materials and Methods. The RU of the immobilized ligands were, CSA: 695, OSCS: 829, and heparin: 814. (**A**) Peak response units (RU) of various chemokines as indicated (at the concentration of 200 nM) binding with immobilized CSA, heparin and OSCS (Biacore sensorgrams are shown in Figure S1). (**B & C**) Biacore sensorgrams are shown for different analyte concentrations (from top 250, 125, 62.5, 31.25, 15.6, 7.8, 3.9 and 1.95 nM) of (**B**) SDF-1α or (**C**) SDF-1β binding to the immobilized GAG surfaces (ligands). The surfaces for the immobilized GAGs were regenerated each cycle using 1 M NaCl plus 50 μM NaOH. The data are representative of three independent experiments.

In order to test if OSCS interacts with chemokines, a group of human CC, CXC and XC chemokines, many of which target T cells, were tested for their binding to OSCS using surface plasmon resonance (Biacore). As shown in Figure 1A and Figure S1, OSCS has a very strong binding to chemokines CXCL10 (IP10), CXCL11 (ITAC) and CXCL13 (BLC); moderate binding to CCL28 (MEC), CXCL12α (SDF-1α) and CXCL9 (MIG); relatively lower binding to XCL1, CCL19(MIP3β), CXCL12β (SDF-1β), CCL18 (MIP4), CXCL16, CCL1 (I309), CCL22 (MDC) and CCL21. In contrast, heparin, used as a control, had low to moderate binding with the tested chemokines. Binding of CSA to chemokines was nearly undetectable. The order of heparin binding to chemokines was IP10>SDF-1α>MIP3β, consistent with a previous report using heparin affinity chromatography [7], [30].

A representative panel of Biacore sensorgrams of different chemokines binding with GAGs is shown in Figure S1. Due to the complexity of binding, deriving meaningful constants from curve fitting these sensorgrams is challenging and we have chosen to compare chemokine binding at one concentration in Fig. 1A. However, a chemokine may have a higher avidity to a GAG even if the RU values at specific concentrations are lower than that of another chemokine. For example, OSCS and heparin bound CCL21 with higher affinities than CCL19/MIP3β (data not shown), using a two-state binding model. This is also visible as a very slow dissociation phase in Figure S1 and is consistent with a previous report by de PaZ et al using a microarray method [31].

In order to study in detail the binding and functional effects of OSCS to chemokines, we focused on two forms of the stromal cell derived factor 1 (SDF-1) chemokine (SDF-1α and SDF-1β), one of the most important and well characterized chemokines. As shown in Fig. 1B, binding of CSA to SDF-1α was minimal, whereas the binding of heparin to SDF-1α was seven times greater than that of CSA. At concentration of 250 nM, SDF-1α binding to heparin was 225 RU as compared with 32 RU for SDF-1α binding to CSA. OSCS showed the greatest binding under the same conditions, 2050 RU, nearly 70-fold that of CSA and ten-fold that of heparin binding with SDF-1α. Similar results for SDF-1β binding with GAGs are shown in Figure 1C. Of note, SDF-1β binding with OSCS is only three fold that of heparin. At 250 nM, SDF-1β binding to OSCS was 460 RU and SDF-1β binding to heparin was 160 RU. SDF-1 binding to GAGs did not fit a 1∶1 stoichiometry or other binding algorithm models, suggesting a complex multivalent binding modality. Both SDF-1α and SDF-1β showed a much slower dissociation from OSCS than from heparin (Fig. 1 B & C), indicating OSCS binds to SDF-1 with a higher avidity than heparin. A two-state binding model also indicates SDF-1β binding to GAGs with higher affinities than SDF-1α (data not shown).

### OSCS Binding to Chemokines Induces Large Conformational Changes

**Figure 2:**
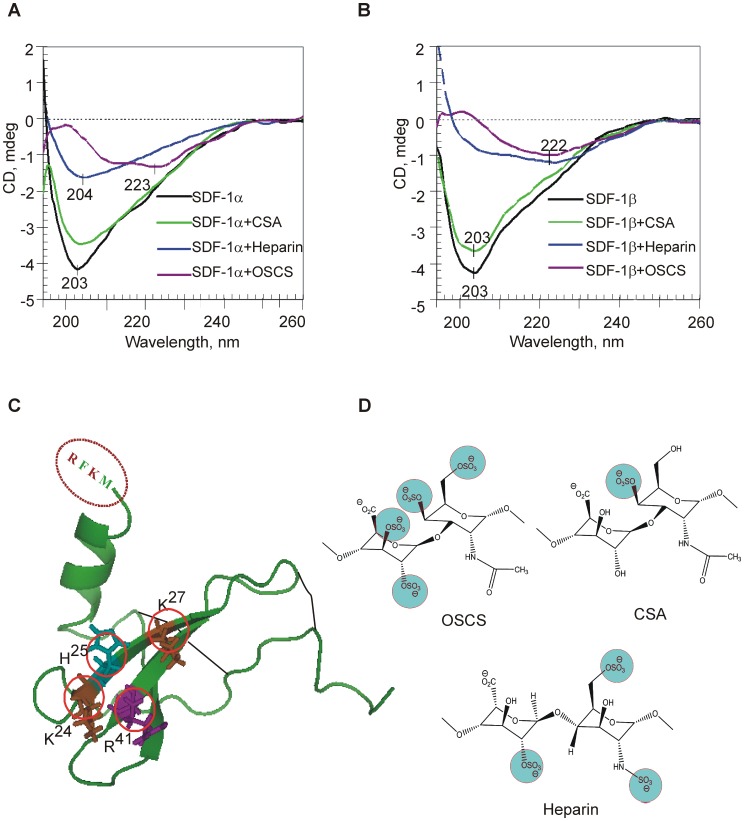
OSCS binding leads to conformational changes of SDF-1. Conformational changes were shown by far-UV CD spectra of 12.5 μM SDF-1α (**A**) and SDF-1β (**B**) in PBS alone (black line), and in the presence of CSA (green), heparin (blue) and OSCS (purple) at the protein: GAG molar ratio of 2∶1. The CD spectra of GAGs (6 μM) alone are negligible (zero line). (**C**) Crystal structure of SDF-1α [PDB code 1SDF] with basic amino acid residues K^24^, H^25^, K^27^, and R^41^ (Hot Spots, in red circles) [48], two disulfide bonds (indicated in black lines), and the four additional amino acid residues present in the C-terminus of the SDF-1β, RFKM. (**D**) The structure of disaccharide repeats of OSCS, heparin and CSA [2], [49]. The negatively charged sulfate units (in blue circles) can bind with positively charged “hot spots” of SDF-1. The highly negatively-charged OSCS is likely to have the best fit with SDF-1 hot spots. The data in A & B are representative of three independent experiments.

In order to understand if there are any potential conformational changes of SDF-1 after binding with GAGs, circular dichroism (CD) spectra of SDF-1 with and without CSA, heparin and OSCS were compared. As shown in figure 2 A & B, the far-UV CD spectra of SDF-1α and SDF-1β are characterized by a major negative extremum at 203 nm, typical for proteins with a high content of β-turns and β-sheets [32], and is consistent with the CD spectra of other chemokines [33]–[35]. Evaluation of the secondary structure of SDF-1α by CD/CONTIN revealed ∼16% α–helix, 33% of β-sheet, 22% β-strands, and 29% unordered structure, comparable with the estimates published earlier [33], [35] and crystal structure data [36]. In the GAG concentration range used in this study (from 6 to 24 μM), CD spectra of the GAGs were negligible. Thus significant spectral changes, induced by these GAGs, reflect large conformational alterations in SDF-1α or SDF-1β. In the presence of CSA, CD spectra of both SDF-1α and SDF-1β showed a ∼13–15% reduction in the band intensity with no significant changes in the CD pattern. With the same amount of OSCS, both SDF-1 chemokine forms showed a very large change of the initial secondary structure as observed by the loss of the 203 nm band and the gain of a broad low-intensity CD band at 222–223 nm, typical for β-sheet dominated structures. Addition of heparin to the SDF-1 chemokines resulted in a significant reduction of the intensity of the band at 203 nm (∼61% and 72% for SDF-1α and SDF-1β respectively), but for SDF-1α the initial CD pattern can still be observed (Fig. 2A). Comparison of CD spectra corresponding to SDF-1α and SDF-1β with OSCS and heparin suggests that conformational alterations in case of SDF-1β are more significant than those observed for SDF-1α (a stronger reduction of the initial CD intensity and a shift to 222 nm). This could likely be due to the presence of four extra amino acid residues at the C-terminus of SDF-1β, including two positively charged amino-acid residues, arginine and lysine (R^69^ and K^71^). The additional positive charges in SDF-1β very likely indicate a higher binding capacity towards negatively charged GAGs and, therefore, different conformational alterations.

An analysis of the structural and functional basis of SDF-1α interaction with disaccharide heparin conducted by Murphy and co-workers revealed two clusters of important residues: (1) H^25^, K^27^, and R^41^, and (2) A^20^, R^21^, N^30^, and K^64^ [37]. Figure 2C illustrates the positively charged amino acid residues on the SDF-1α monomer, whereas SDF-1β possesses two extra positively charged residues. The difference in SDF-1 binding patterns among CSA, heparin and OSCS might be due to their differing numbers and arrangements of negative charges interacting with the positively charged amino acids (hot spots) in SDF-1. The highly negatively charged OSCS provides the best match (Fig. 2D). SDF-1α as well as SDF-1β with its additional positive charges, allow for alternative and/or multiple binding sites in GAG disaccharides. Larger chains of GAGs may interact with a larger number of SDF-1 molecules.

### OSCS Inhibits SDF-1-mediated T cell Chemotaxis

**Figure 3:**
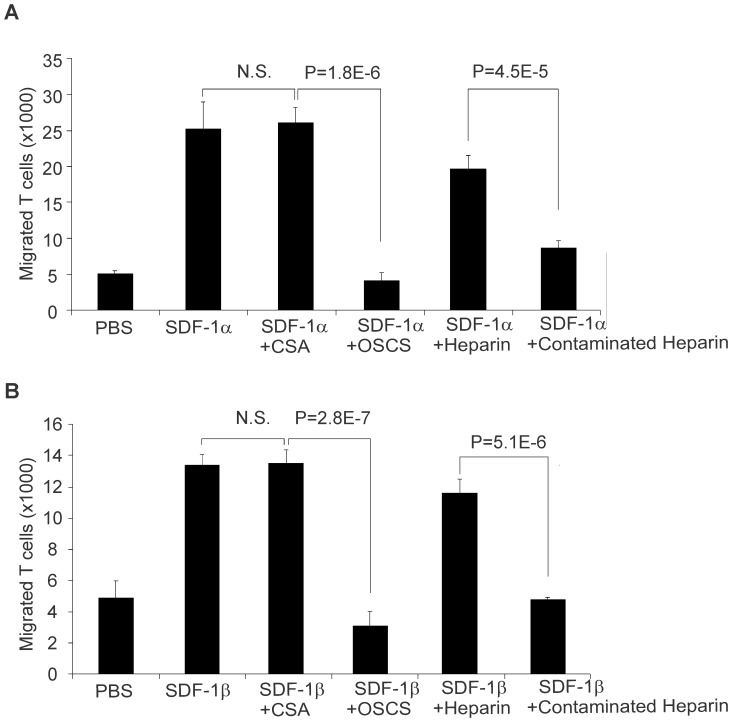
OSCS inhibits SDF-1-mediated T cell chemotaxis. PHA and IL-2-induced human T cell blasts were added at 10^5^/100 μL per well to the upper chambers of 24-well transwell plates. 1 mL serum-free culture medium with 100 ng recombinant SDF-1α (**A**) or SDF-1β (**B**), alone or in the presence of 25 μg/ml of CSA, OSCS, heparin or a heparin lot contaminated with OSCS were added to the lower chambers as described in the Materials and Methods section. After two hours of incubation at 37°C in a CO_2_ incubator, the cells that migrated to the lower chambers were counted by flow cytometry using BD Trucount tubes. P values of one-way ANOVA test among groups were less than 0.0001 for both (A) and (B) supporting the hypothesis that the population means are not all equal. P values based on Student’s t test of the differences between SDF-1 with CSA and SDF-1 with OSCS and between SDF-1 with heparin and SDF-1 with contaminated heparin are shown. There is no significant difference between SDF-1 and SDF-1 with CSA. The data are the averages of triplicates and the experiment has been repeated with cells from at least three different donors.

To test the functional effects of SDF-1 after binding with OSCS, we choose PHA stimulated blast T cells as a model. A previous study showed that SDF-1 was more potent for receptor-stimulated T cells than for freshly isolated cells [29] and a more potent chemoattractant than other CXC or CC chemokines for receptor-stimulated T cells. As shown in Fig. 3, 80 ng/ml (10 nM) SDF-1α added to the lower chamber of a transwell induced migration of a large number of blast T cells. In the presence of 25 μg/ml OSCS, this SDF-1α induced chemotaxis was totally blocked. As a control for OSCS, the same concentration of CSA did not affect SDF-1α induced chemotaxis. As expected from the binding data, heparin at the same concentration (25 μg/mL) had mild (∼20%) inhibition of SDF-1α induced T cell migration. However, a heparin lot contaminated with OSCS led to much stronger inhibition of SDF-1α mediated cell migration than uncontaminated heparin (Fig. 3A). A lower concentration of GAGs, 5 μg/mL, resulted in a similar pattern but less dramatic inhibition (data not shown). The impact of GAGs on SDF-1β-induced T cell chemotaxis has a similar pattern to that of SDF-1α (Fig. 3B).

### OSCS Inhibits SDF-1-mediated T cell Calcium Mobilization

**Figure 4:**
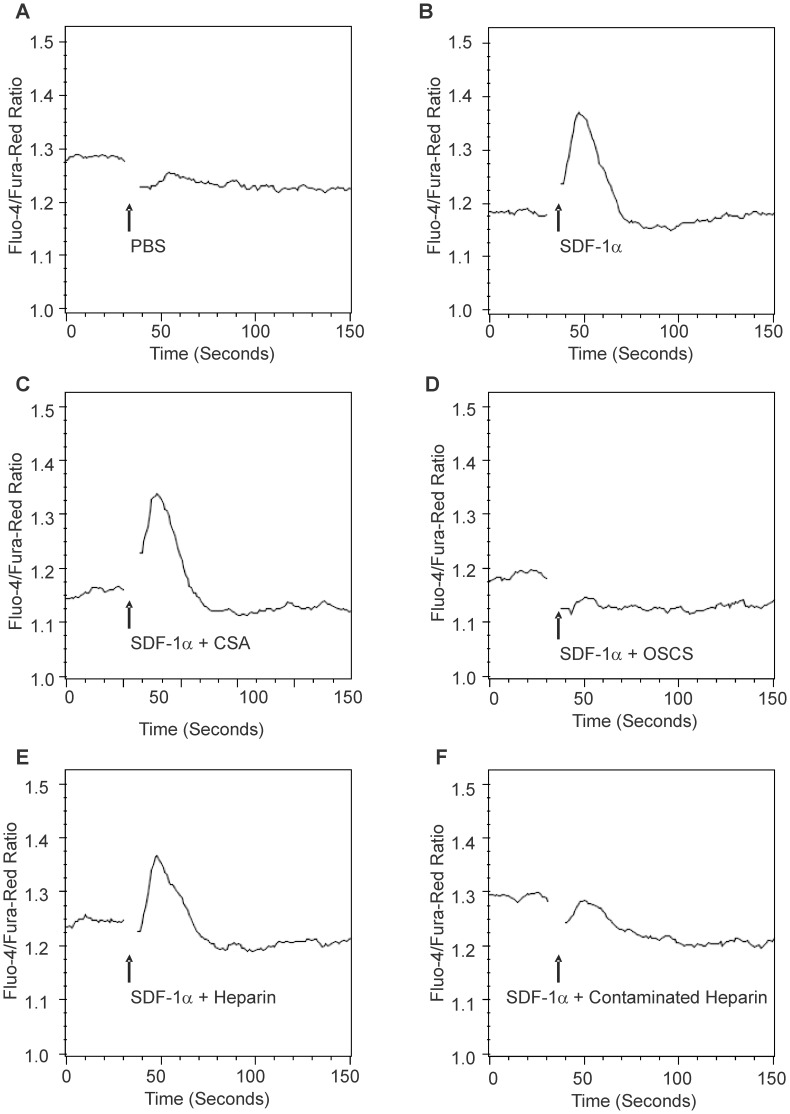
OSCS inhibits SDF-mediated T cell calcium mobilization. PHA and IL-2 activated human T cell blasts were loaded with fluo-4 and fura red dyes as described in the Materials and Methods section. The T cell response to SDF-1α was detected using a LSRII flow cytometer and expressed as the fluorescence ratio of fluo-4/fura-red dyes. (**A**) The injection of PBS did not cause a calcium influx in T cells; (**B**) 100 nM SDF-1α induced a strong calcium influx in T cells; (**C**) SDF-1α in the presence of CSA induced a strong calcium influx in T cells; (**D**) SDF-1α in the presence of OSCS did not cause a calcium influx in T cells; (**E**) SDF-1α in the presence of heparin induced a calcium influx in T cells; (**F**) SDF-1α in the presence of heparin contaminated with OSCS did not induce a calcium influx in T cells. The data are representative of experiments with at least three independent donors.

Binding of SDF-1 to G-protein-coupled receptor CXCR4 activates heterotrimetric G-proteins such as Gαi and Gαq, triggers serial signal events involving inositol triphosphate (IP3) and diacylglyerol (DAG), and leads to calcium mobilization and chemotaxis [38]. As shown in Fig. 4B, SDF-1α induced a strong calcium influx in PHA-activated T cells. OSCS completely inhibited this SDF-1α-induced calcium mobilization (Fig. 4D) as compared to the CSA control which did not affect SDF-1α-induced calcium influx (Fig. 4C). We did not observe a noticeable inhibition of calcium mobilization by heparin at the concentration of 25 μg/mL (Fig. 4E). However, a heparin lot contaminated with OSCS markedly inhibited SDF-1α-induced calcium mobilization (Fig. 4F). Interestingly, while SDF-1β showed a similar pattern to that of SDF-1α in the T cell chemotaxis experiment, uncontaminated heparin inhibited calcium influx by SDF-1β (Figure S2) without inhibiting SDF-1α-induced calcium influx. This differential effect could likely be due to the larger impact of heparin on SDF-1β structure as shown in the CD spectra results (Fig. 2A and B).

### OSCS Inhibits SDF-1-induced T cell Morphological Changes

**Figure 5:**
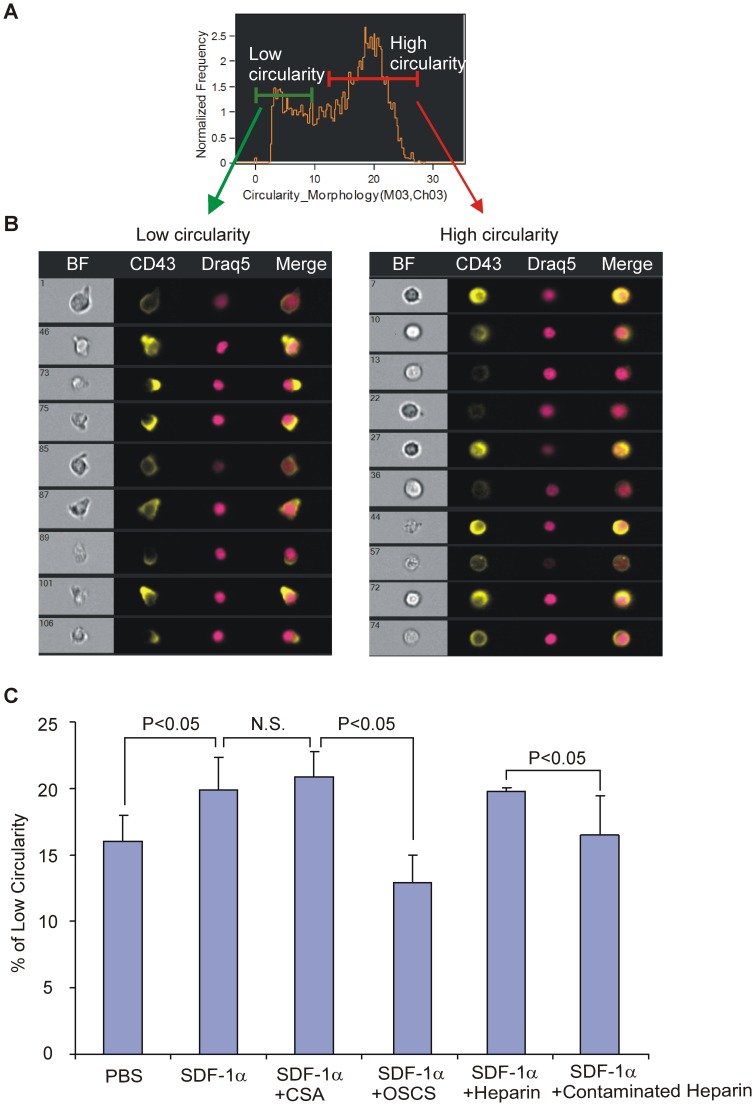
OSCS inhibits SDF-1α mediated T cell morphological changes. PHA and IL-2 activated human T cell blasts were stained with anti-CD43-FITC for the CD43 membrane distribution, Draq5 for the nucleus and 7AAD to exclude dead cells as described in the Materials and Methods section. Analysis by ImageStream of T cells in response to SDF-1α with cells gated into low circularity and high circularity populations were (**A**) based on the membrane Circularity_Morphology histogram; (**B**) Representative images showing low circularity and high circularity cells; (**C**) Percentages of low circularity cells in the whole cell population after treatment with PBS, SDF-1α, SDF-1α in the presence of CSA, OSCS, heparin or heparin contaminated with OSCS. There is no significant difference between SDF-1α and SDF-1α in the presence of CSA (0.1>p>0.05). A significant difference exits between samples treated with PBS and SDF-1α (0.02>p>0.01), SDF-1α with CSA and SDF-1α with OSCS (0.02>p>0.01) as well as between SDF-1α with heparin and SDF-1α with contaminated heparin (0.02>p>0.01). The data are representative of experiments with at least three different donors.

Binding of SDF-1 to its receptor CXCR4 can regulate the cytoskeleton and induce actin polymerization and morphological changes via GTPase signaling pathways. It has been previously shown that movement of T-cell surface CD43 is associated with both antigen-driven activation and chemokine-induced T cell motility [39]. Imaging flow cytometry was used to analyze the morphological changes in PHA-stimulated T cells incubated with SDF-1α in the absence or presence of GAGs. The cells were gated into two populations based upon membrane CD43 movement - a low circularity population and a high circularity population (Fig. 5A). As shown in Fig. 5B, high-circularity cells exhibited fewer protrusions, while the low-circularity cells were bell-shaped and polarized. Calculation of the percentage of low circularity cells allowed us to quantify the degree of cell morphological change induced by SDF-1α and the effect of GAGs on this change. As shown in Fig. 5C, PHA-activated T cells had a baseline level of polarized (low circularity) cells, and after addition of SDF-1α, the percentage of polarized cells significantly increased. The presence of OSCS reduced SDF-1α-induced cell morphological changes to the baseline level. As a control, CSA had no effect on SDF-1α-induced polarization of T cells. Uncontaminated heparin, at 20 μg/mL, had minimal inhibition of SDF-1α-induced cell shape change. However, OSCS-contaminated heparin caused a significant reduction of the polarized cells.

## Discussion

In the present report, using surface plasmon resonance, we show that OSCS has strong binding with chemokines and may therefore influence the regulation of the immune system through modulating chemokine-driven functions, such as immune cell migration. We then focused on the interaction of OSCS with SDF-1 chemokines.

SDF-1 is a chemoattractant for monocytes and lymphocytes and exerts its activity through binding with the chemokine receptor CXCR4 [18] and activation of heterotrimeric G-protein (Gαi, Gαq and Gβγ) pathways [38]. These pathways mediate cell migration, transcriptional activation, cell growth and differentiation [40]. To determine whether the interaction of OSCS with SDF-1 altered SDF-1-CXCR4-mediated signaling in T cells, three different cell behaviors associated with different downstream signaling pathways were examined. In SDF-1-CXCR4 signaling, calcium influx and chemotaxis pathways split after PIP2 hydrolysis in the Gαq-mediated pathway. Both of these downstream pathways are independent from the actin cytoskeleton signaling pathway which starts from GTP-bound Gβγ [38]. Our demonstration of OSCS-dependent inhibition of all three independent downstream pathways indicates that OSCS blocking of SDF-1-CXCR4 signaling may have impacted very proximal events - a likely candidate being the binding of SDF-1 with CXCR4. Of note, in stimulation of freshly isolated T cells through a TCR-mediated signaling pathway (using PHA), calcium influx and T cell survival were not affected by the presence of OSCS (data not shown). Thus a direct impact of OSCS on T cells is unlikely.

However, SDF-1 possesses a unique GAG binding cluster (cluster 2), with a very clear separation between the receptor (N loop) and the GAG (β1 and β2 strands) binding sites, which are localized on the opposite sides of the molecule [41]. Thus it is not clear that GAGs directly block the binding of chemokines to their receptors. Our demonstration of SDF-1α binding to CSA or heparin, without affecting SDF-1-dependent signaling in activated T cells, supports the notion that GAG binding sites and chemokine receptor binding sites are structurally distinct and do not sterically interfere with each other [41], [42].

In order to determine why OSCS but not CSA affected SDF-1 mediated signaling, and whether it was related to alteration in the secondary structures, we performed circular dichroism experiments. The results from these studies demonstrated that the binding of OSCS with SDF-1 led to dramatic changes in the conformational structure of SDF-1. Based on these observations, it is reasonable to hypothesize that the structural changes induced by OSCS binding could alter the SDF-1 receptor binding site and prevent receptor binding or lead to ineffective SDF-1-CXCR4 interactions.

During normal physiology, cell-surface GAGs with low levels of sulfation, such as heparan sulfate (HS), can enhance chemokine immobilization. By forming haptotactic gradients, cell-surface GAGs can also influence chemokine transport, clearance, degradation, and oligomerization [43]. Without such an immobilization mechanism, chemokine gradients would be disrupted by diffusion [7]. Thus, HS –chemokine interactions may control the migration of specific populations of cells and determine which leukocyte subsets enter tissues [31], [43]. In this scenario, soluble GAGs with higher avidity to chemokines than HS, such as heparin, could break the HS-chemokine gradients causing leukocytosis [44]. Interfering with chemokine gradients may also block leukocyte-mediated inflammation by altering the trafficking of inflammatory immune cells [43] or block tumor cell migration [45]. However altering the chemokine gradient without blocking chemokine signaling may only have a limited effect on inflammation or tumor metastasis. OSCS, by dramatically changing the conformational structure of chemokines, may completely shut down the signaling of multiple chemokines and therefore block cell migration more efficiently than heparin.

Heparin lots contaminated with OSCS can also inhibit SDF-1α associated signaling and SDF-1 driven T cell migration. Patients exposed to contaminated heparin by intravenous dosing are unlikely to have sustained levels associated with this inhibition although subcutaneous administration [46] of contaminated heparin may have allowed for higher local levels of OSCS and a clinically meaningful impact on chemokine mediated inflammatory responses.

The clinical development of heparin as an anti-inflammatory drug has been hampered by many potential side effects including thrombocytopenia [47] and undesired anti-coagulant activity. While OSCS may not have the same potent anticoagulant activity as heparin, it can induce contact system activation. Therefore the design of GAGs (e.g. by controlling length and sulfation) that are able to inhibit chemokines without anti-coagulant activity or kallikrein activation may lead to the development of novel therapeutics for controlling chemokine-mediated inflammation, tumor metastasis, and other related medical conditions.

## Conclusions

Many glycosaminoglycans (GAGs) including oversulfated chondroitin sulfate (OSCS), a heparin contaminant linked to severe adverse events in 2007–2008, bind to chemokines. OSCS had the strongest binding and blocked SDF-1 induced signaling in activated T cells. Thus OSCS can inhibit mediators of adaptive immunity, chemokines, as well as mediators of inflammation. OSCS binding was associated with a large conformational alteration of SDF-1 chemokines. The structural features associated with these effects suggest approaches for developing novel immunomodulatory therapeutics as well as providing additional information on the potential adverse effects of GAGs and related molecules.

## Supporting Information

Figure S1 (TIF)

Figure S2 (TIF)
