# Supplementary figures and images for: Oversulfated Chondroitin Sulfate Binds to Chemokines and Inhibits Stromal Cell-Derived Factor-1 Mediated Signaling in Activated T Cells
Source: PLoS One. 2014 Apr 9;9(4):e94402. doi: 10.1371/journal.pone.0094402 (PMC3981801; doi:10.1371/journal.pone.0094402)

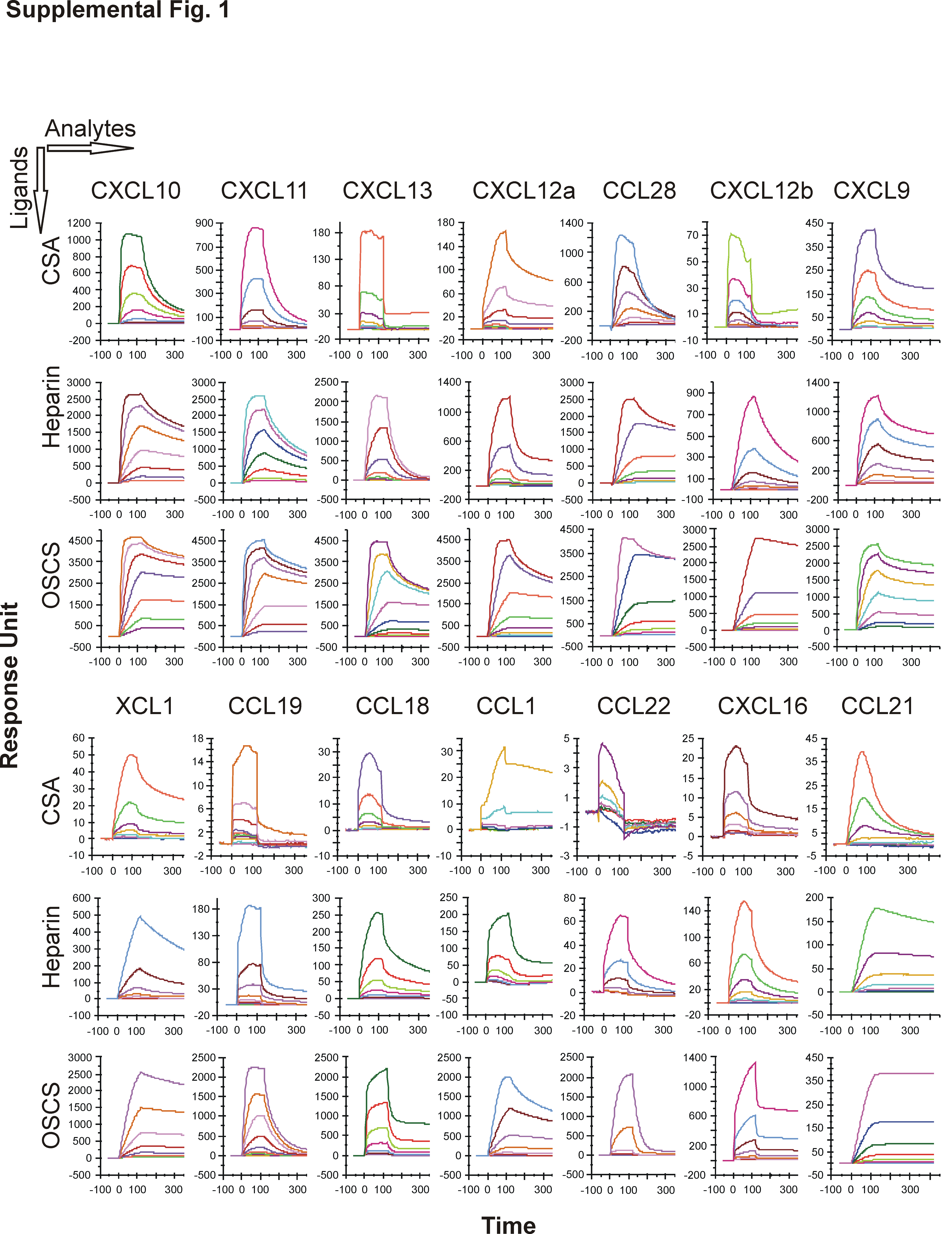

Supplement: Figure S1 — (TIF) [file pone.0094402.s001.tif]

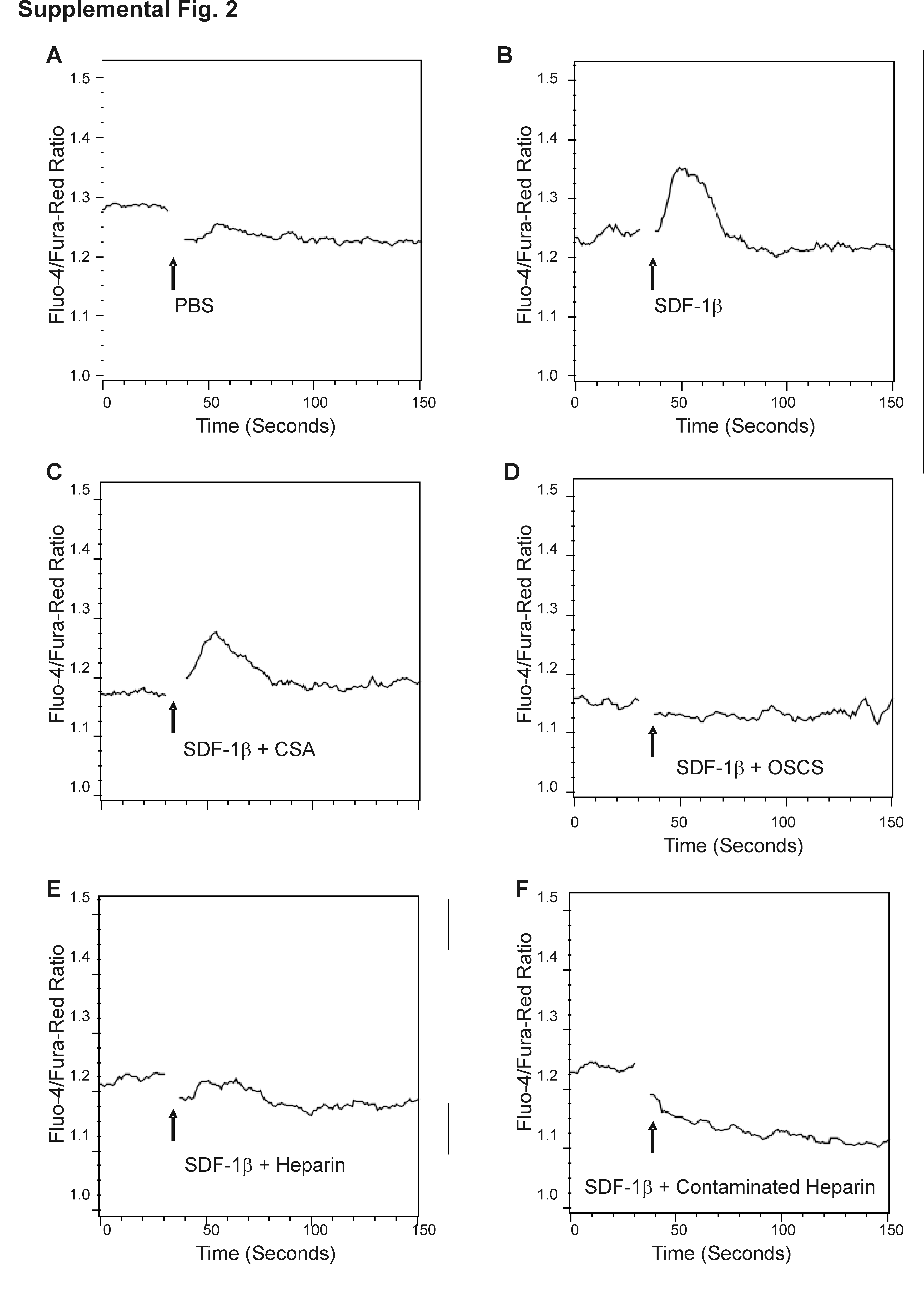

Supplement: Figure S2 — (TIF) [file pone.0094402.s002.tif]
